# Direct and Indirect Role of Migratory Birds in Spreading CCHFV and WNV: A Multidisciplinary Study on Three Stop-Over Islands in Italy

**DOI:** 10.3390/pathogens11091056

**Published:** 2022-09-16

**Authors:** Elisa Mancuso, Luciano Toma, Ilaria Pascucci, Silvio Gerardo d’Alessio, Valeria Marini, Michela Quaglia, Sara Riello, Andrea Ferri, Fernando Spina, Lorenzo Serra, Maria Goffredo, Federica Monaco

**Affiliations:** 1 Istituto Zooprofilattico Sperimentale dell’Abruzzo e del Molise ‘G. Caporale’, 64100 Teramo, Italy; 2 Department of Biomolecular Science, University of Urbino “Carlo Bo”, 61029 Urbino, Italy; 3 Istituto Superiore di Sanità, 00161 Rome, Italy; 4 Istituto Zooprofilattico Sperimentale dell’Umbria e delle Marche “T. Rosati” Sezione Di Pesaro, Via dei Canonici 140, 61122 Pesaro, Italy; 5 Riserva Naturale Statale “Isole di Ventotene e S. Stefano”, 04031 Ventotene (LT), Italy; 6 Area Avifauna Migratrice, Istituto Superiore per la Protezione e la Ricerca Ambientale (ISPRA), 40064 Ozzano dell’Emilia, Italy

**Keywords:** vector-borne viruses, zoonoses, CCHFV, WNV, migratory birds, Italy

## Abstract

The annual movements of migratory birds can contribute to the spread of African ticks and tick-borne pathogens of potential public health concern across Europe. The aim of the study was to investigate their role in the possible introduction of African ticks and tick-borne pathogens into European countries during spring migration. A total of 2344 ticks were collected during three spring seasons from 1079 birds captured on three Italian stop-over islands during their northbound migration. Once identified, each tick was tested by RT-PCR for the presence of Crimean-Congo hemorrhagic fever (CCHFV), West Nile (WNV), and Usutu (USUV) viruses. Moreover, carcasses of birds found dead were collected and tested for the possible presence of WNV and USUV. Results confirmed a higher contribution of trans-Saharan migrants compared to intra-Palearctic ones and the prevalence of African tick species in the sample. CCHFV was detected for the second time in Italy in a *Hyalomma rufipes*, and WNV was found in two ticks of the same genus, all carried by trans-Saharan birds. WNV lineage 1 was also found in the organs of a Garden warbler. These results confirm the role of migratory birds in carrying African ticks, as well as viruses of zoonotic importance, from Africa into Europe.

## 1. Introduction

Climatic and environmental changes influence complex ecological systems where pathogens, vectors, hosts, habitats, and landscapes interact [1]. Increasing temperatures and changes in the ecosystems due to human activities support the development, survival, increasing density, and areal expansion of arthropod vectors and, consequently, the risk of vector-borne zoonoses, which represent a threat to human and animal health worldwide. Ticks are among the most common biological and mechanical vectors of viral and bacterial diseases, and they have an extremely wide range of distribution despite their limited ability in movement. Nevertheless, they can be easily moved far from their original range thanks to their hosts. Due to the unique ability to fly for long distances between continents in a short lapse of time and to cross the most majestic geographical barriers, migratory birds can move pathogens, acting themselves as reservoirs, or carry ticks and related pathogens, promoting the spread along their routes [2]. This migrants-related origin has been investigated for re-emerging zoonotic pathogens strictly dependent on bird hosts [3] circulating in Europe in the last decades, such as the Usutu virus (USUV) and West Nile virus (WNV). This last virus is present in the continent with two of the several known lineages that have been associated with significant outbreaks in humans and horses as a result of possible different repeated introduction events related to bird movements [4]. Likewise, birds are considered to have a role in disseminating ticks and tick-borne pathogens out of their endemic areas, including the Crimean-Congo hemorrhagic fever virus (CCHFV), the etiological agent of one of the priority diseases requiring urgent research and development efforts according to the World Health Organization [5,6]. CCHFV, genus *Orthonairovirus* within the Nairoviridae family, is the most geographically widespread tick-borne viral pathogen affecting humans with a high fatality rate, while other infected mammal hosts develop a short-term viremia with no visible symptoms. Viremia does not develop in most passerine birds, which are not able to pass the virus to ticks [7,8].

Evidence of an active role of migratory birds in the diffusion of CCHFV-infected ticks has been obtained by direct captures and bird screening in countries where the virus is endemic, such as Morocco [9], Turkey [10], and Greece [11]. Furthermore, the CCHFV genome has been recently detected in a tick collected from a bird in Italy as a preliminary result of this research project [12]. CCHFV is endemic in sub-Saharan Africa, Asia, Turkey, and Balkan Peninsula in Europe, with at least six different known genotypes. It was not present in western Europe until 2010, when the virus, belonging to the African genotype III, was found for the first time in Spain in a tick collected from a red deer [13]. After some years with new detections in ticks on wild animals and few human cases, the virus is now considered endemic in Spain, circulating in wildlife through tick transmission [14]. The genotype characterization suggested a viral introduction directly from Africa, which can likely be attributed to the passive transport of infected ticks from regions with viral circulation. Furthermore, the active role of migratory birds in spreading exotic ticks has also been confirmed by numerous findings of adult stages of typical sub-Saharan ticks, such as *Hyalomma rufipes*, in Spain [15], Netherland [16], Hungary [17], Germany [18,19], Great Britain [20], Sweden [21], Czech Republic [22,23], and *Amblyomma variegatum* in Corsica, France [24], and Italy [25] as possible results of immature stages seeded by avian hosts, molted once on the ground and survived to the new environmental conditions. Moreover, some of the mentioned specimens of *H. rufipes* were found positive for *Rickettsia aeschlimannii* [19,20,21], and both the *A. variegatum* ticks were positive for *Rickettsia africae*, hence supporting their potential role in tick-borne pathogen introduction. Transport of immatures of exotic ticks by birds captured in Italy during migration has been largely confirmed in the last years [26,27,28,29,30]. This issue suggests the need for strengthening vector and veterinary surveillance as also supported by recent prediction models that highlight the risk of CCHF emergence in Italy due to the new climatic and environmental conditions, the presence of endemic competent vector species, and its location in the middle of the Mediterranean area [31]. The Italian peninsula is indeed in the center of one of the most important bird migration flyways connecting Africa to Europe, and it is overflown every year by billions of birds belonging to hundreds of species that land to reach their breeding grounds on the territory or just temporarily before continuing their northbound journey. Most of those species are trans-Saharan or long-distance migrants breeding in Europe and spending the rest of the year in sub-Saharan areas, whereas intra-Western Palearctic or short-distance migrants, after breeding in Europe as well, do not cross the Saharan desert.

Thus, on the example of the other pilot studies in the Mediterranean Basin [28,30,32], we conducted an extensive three-year study by collecting ticks from birds and free-living ticks on the stop-over islands during the spring migration seasons. The aim was to evaluate the role of migratory birds, particularly of trans-Saharan migrants, in spreading exotic ticks and related viruses, focusing on CCHFV. In addition, presence of WNV and USUV was investigated in bird carcasses but also in ticks, despite they are not competent vectors, to indirectly detect the viraemia in bird hosts as reservoirs of these two viral zoonotic agents.

## 2. Materials and Methods

### 2.1. Samples’ Collection

**Figure 1:**
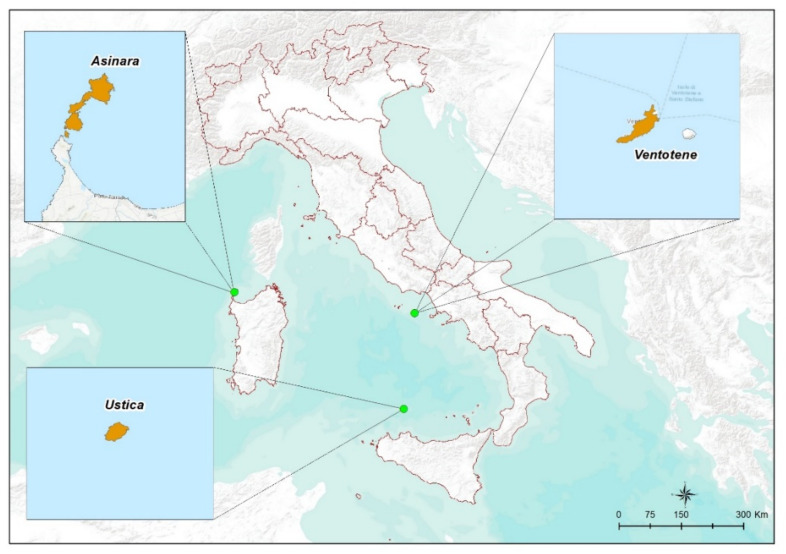
Sampling sites on stop-over islands during spring bird migration from Africa.

In the present study, tick sampling was carried out during the spring seasons 2017, 2018 and 2019 on three different islands of the Tyrrhenian Sea in Italy: Ventotene (area: 1.54 km^2^), Asinara (area: 50.9 km^2^) and Ustica (area: 8 km^2^) (Figure 1). These islands are known to be important temporary stop-over sites for birds during the spring migration from Africa on the way northward to breeding quarters, and for this reason, they host ornithological observatories carrying on bird-ringing activities, allowing the screening for the presence of parasites during the annual catches. Ventotene island, in particular, the smallest among the three, is characterized by an extraordinarily intensive passage of birds since it is the first stop-over site for thousands of migrants after a non-stop flight over the sea from Northern African coasts [33]. Once on the island, birds spend only few hours up to few days to rest and feed before leaving [34].

The sampling sessions during 2017 and 2018 covered the entire period of activity of each ringing station (8 March–30 May in Ventotene, 1 April–15 May in Asinara, and 15 April–15 May in Ustica), whereas in 2019 sampling was limited to the island of Ventotene and to a shorter period (from 9 April to 30 May) to focus the research exclusively on African ticks and pathogens during the passage of trans-Saharan migratory birds. In all the localities, birds were captured every day from one hour after the first daylight to one hour after dark according to weather conditions, using mist-net transects (32 nets for a total of 351 m in Ventotene; 28 nets for 324 m in Asinara; and 25 nets for 300 m in Ustica). Once caught, birds were identified at species level, aged and sexed when possible, subjected to measurements, weighted, inspected for presence of ticks and finally released. All procedures, included screening for parasites and their collection, were performed by authorized bird-ringers and trained personnel. Ticks were removed by using plastic Tick Twisters (O’Tom) or stainless-steel tweezers (Dumostar #3),preserved at room temperature in 2 mL Eppendorf tubes containing 70% ethanol and molecularly processed within 4 months from the collection. All the parasites picked up from each single bird were stored together in the same tube, recording the collection date and host species. In 2017 and 2018, simultaneously with bird-ringing activities, few random attempts of free-living ticks’ collection were made through the dragging method by using a 1 square meter white cotton cloth. These samplings were finalized to investigate the possible introduction of African species and viral circulation among vectors on the stop-over islands.

Moreover, a total of 101 carcasses of birds found dead for natural causes in 2017 and 2019 were collected in Ventotene island and necropsies were practiced to excise brain, heart and kidneys, to test the possible presence of WNV and USUV RNAs by molecular analysis. Organs were collected in 1.8 mL cryotubes, kept in a portable liquid nitrogen tank and then stored at −80 °C once in the laboratory.

### 2.2. Statistical Analyses

Bird species were divided according to the main migratory strategies: long-distance migrants (LD) including birds breeding in Europe and wintering in sub-Saharan Africa; short-distance migrants (SD) including intra-Western Palearctic species, moving within the northern hemisphere and reaching northern Africa as the most southern limit of their range during the Boreal winter season; and resident species (R) which are sedentary [35]. A chi-square test was performed to evaluate the difference in the overall infested birds, number of ticks within the sample, and prevalence of infested birds (Prevalence = number of infested birds/number of screened birds) between the two main migratory strategies (LD vs. SD)in order to estimate their contribution in the potential introduction of ticks in the peninsula over the whole three-year period. 

### 2.3. Tick Identification, Nucleic Acids Extraction, and Pathogens’ Detection 

After collection, the ticks were morphologically identified by using a stereomicroscope following the dichotomous keys described by Manilla [36] and Iori et al. [37]. Tick species included in the checklist of the Italian ticks [36,37] were considered autochthonous and allochthonous all those not included. Some species present in North Africa are also reported in the checklist, but it is not possible to distinguish the geographical origin of the individuals, whether in North Africa or in the Italian Mediterranean islands.

Being impossible to identify species in immature stages, most of larvae and nymphs were further processed for molecular identification. As described by Toma and colleagues [29], following the morphological and molecular identification results obtained in 2017, only a sample made by about half of the numerous immature ticks, mainly *Hyalomma* spp. and *Ixodes* spp., was molecularly identified in 2018 and 2019, respectively. 

Ticks were taken out of 70% ethanol, air dried, and homogenized singularly in the lysis buffer provided by the extraction kit, using a 5 mm steel bead and the Tissue-lyser LT (Qiagen, Hilden, Germany). In 2017 nucleic acids were extracted from ticks using Maxwell 16 LEV simply RNA blood kit (Promega, Madison, Wisconsin, USA), whereas in 2018–2019 we used the BioSprint 96 One-For-All Vet Kit (Qiagen, Hilden, Germany) following the protocol for animal tissue homogenates. The two chosen kits allow to purify both DNA and RNA and were used according to each manufacturer’s protocols. Organs of birds were homogenized in 1:10 dilution of PBS (phosphate-buffered saline) with antibiotics and centrifugated at 2000× *g* for 10 min. RNA from organs was purified using the BioSprint 96 One-For-All Vet Kit with the same protocol used for ticks.

The presence of viral targets was investigated with specific real-time RT-PCR assays: ticks were individually tested for CCHFV, WNV, and USUV, while birds’ organs were individually tested for WNV and USUV only. In 2017 molecular test for detection of CCHFV RNA in ticks was executed by using the commercial assay RealStar CCHFV RT-PCR Kit 1.0 (Altona Diagnostics, Hamburg, Germany) according to the manufacturer’s instructions. Instead, in 2018 and 2019, for the same screening was used the assay described by Wölfel et al. [38] and positives were further confirmed with the above-mentioned commercial kit. All ticks and organs were tested singularly for WNV using a specific protocol for lineages 1 and 2 [39] and a protocol for all lineages [40], and for USUV according to Cavrini et al. [41].

## 3. Results

### 3.1. Samples’ Collection

During the ringing activities, more than 48,500 birds belonging to 129 species were caught and inspected for ticks but the presence of these ectoparasites was limited to 43 bird species representing more than 45,000 ringed birds. In this study, we will focus on these 43 bird species, which, in our opinion, more actively contribute to the potential spread of exotic ticks and pathogens. In three years, a total of 2344 ticks were collected from 1079 birds. 

### 3.2. Infested Bird Species

**Figure 2:**
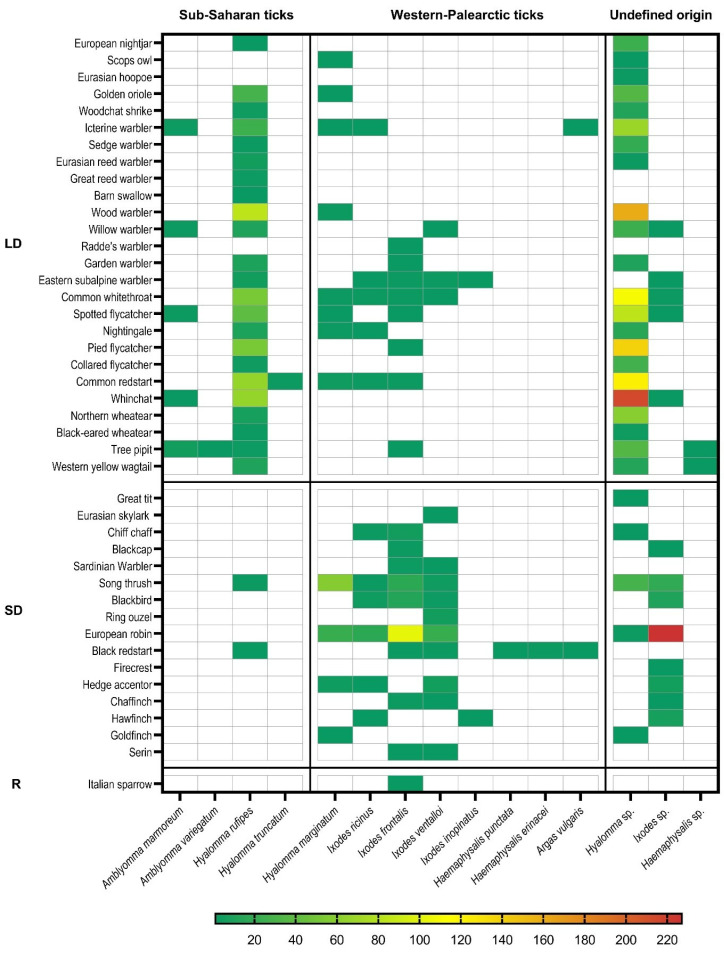
Association between birds’ species and ticks’ species. LD = long-distance migrant, SD = short-distance migrant, R = resident.

Among the 43 parasitized avian species, 40 belonged to Passeriformes order (n = 1061; 98.33%) and 3 to other different orders (n = 18; 1.66%). According to the migratory behavior, 26 species were LD migrants (n = 805; 74.60%), 16 were SD migrants (n = 273; 25.30%), and 1 is a R species (n = 1; 0.09%) (Table 1). The number of ticks per bird ranged between a minimum of 1 and maximum of 55, with a mean of 2.19 ticks per individual. Bird species resulted to be most frequently infested by ticks among LD migrants were: Common redstart, Whinchat, Golden oriole, Western yellow wagtail (Table 1), which are mainly vehicles of sub-Saharan African ticks (Figure 2, Table S1). At the same time, the highest abundance of ticks (number of all ticks parasitizing a bird species) was registered for the following LD migrants: Northern wheatear, Whinchat, and European nightjar (Table 1). The top tick-infested SD migrants were Blackbird, Song thrush, European robin, and Chaffinch (Table 1) which also had the highest abundance of ticks. In contrast, these species were infested mainly by Western Palearctic ticks (Figure 2, Table S1). The species with a sample of fewer than 50 individuals screened were ignored in this analysis.

### 3.3. Statistical Analysis: Migratory Strategies and Spread of Ticks 

Regardless of the species in the totality of the sample, the contribution of LD migratory birds was overall significantly higher both in terms of parasitized birds (74.70% vs. 25.21%, *p* < 0.01) and in the number of ticks (73.72% vs. 26.24%, *p* < 0.01) compared to the SD migrants. However, comparing the prevalence of infestation among the two groups (Prevalence LD = 2.13%, Prevalence SD = 3.83%), the difference is still significant, but the highest contribution is provided by SD migrants (*p* < 0.01). Resident bird species were representative of a minority among infested species contributing only 0.09% of total infested birds and 0.04% of total ticks collected. 

### 3.4. Tick Identification: Species Introduced to Europe with Migratory Birds

Ticks feeding on birds were mostly immature (98.25%), at different degrees of engorgement, consisting of 1385 nymphs (59.09%), 918 larvae (39.16%), and only 41 adults (1.75%). All the specimens were represented by hard ticks (Ixodidae), except for two soft ticks (Argasidae) belonging to the species *Argas vulgaris*. Morphological traits identified *Hyalomma* as the most abundant genus among the collected arthropods, followed by *Ixodes*, *Amblyomma*, *Haemaphysalis,* and *Argas* genera. Details of bird and tick species association are shown in Figure 2, and raw data are reported in Supplementary Materials (Table S1). 

Considering exclusively ticks identified at the species level (n = 855), SD migrants carried more frequently (93,84% within the group) autochthonous tick species, mostly belonging to the *Ixodes* genus (*I. frontalis*, *I. ricinus*, *I. ventalloi*, *I. inopinatus*) which is the most abundant of the Mediterranean area, while only a small percentage was represented by *Hyalomma* ticks, mainly identified as *H. marginatum*. Conversely, LD migrants, were mainly infested (>90% within the group) by allochthonous *Hyalomma* species. Among this genus, molecular identification of immatures, evidenced the preponderant presence of *H. rufipes* (98.61% of examined *Hyalomma*). This sub-Saharan species, was the most represented during the three years, always associated with trans-Saharan migrants with the only exception of two nymphs infesting a Song thrush (SD). The few sub-Saharan *Amblyomma* ticks were found exclusively on trans-Saharan migrants, whereas two soft ticks *Argas vulgaris* were found respectively on a Icterine warbler (LD) and on a Black redstart (*Phoenicurus ochruros*, SD). Finally, the contemporary presence of two different tick genera and species on the same host was reported on a Yellow wagtail (*Motacilla flava*) and a Tree pipit (*Anthus trivialis*), both LD migrants parasitized by a *H. rufipes* and a *Haemaphysalis* sp., not identifiable at species level even through the molecular tests. Searching for free-living ticks, led to collect a total of 337 tick specimens only in Asinara and Ustica in 2017 and 2018, all adults identified by morphology and belonging to autochthonous tick species as described in the study by Toma et al. [29].

### 3.5. Pathogens’ Detection in Ticks

As a result of the screening for viral targets in ticks, CCHFV RNA was found in two immature *H. rufipes* parasitizing birds captured in Ventotene island: one nymph collected in April 2017 from a Whinchat (*Saxicola rubetra*) (GeneBank access numbers: MH595794, MH576575), and a larva collected in May 2018 from a Black-eared wheatear (*Oenanthe hispanica*) (sequencing failed), both trans-Saharan migratory species. The two birds hosted other feeding ticks that resulted negative for the same viral target. Moreover, RNA of WNV lineage 1 was found in an *H. marginatum* nymph collected from a Song thrush in Ventotene in 2017 (Ct-value 38) and in a nymph identified as *Hyalomma* sp. collected from a Tree pipit on the same island in 2018 (Ct-value 37). 

### 3.6. Pathogens’ Detection in Birds

Among the carcasses tested, WNV Lineage 1 was detected in the heart (Ct-value 29), and kidneys (Ct-value 28) of a Garden warbler (*Sylvia borin*) found dead in 2019 on Ventotene Island. All the organs were instead negative for USUV.

## 4. Discussion

Understanding the contribution of migratory birds in moving pathogens, both directly as reservoirs and indirectly by carrying infected ticks, is crucial to assess the risk of their introduction and spread in new areas. In this manuscript, we provide the results, as cumulative data, regarding the prevalence of the most represented tick genera and the association with the different avian species. Results of this extensive and long-term research highlight how the migratory strategy strongly influences the bird–tick species association and the diffusion of allochthonous tick species in the central Mediterranean region. In our sample, which we consider to be representative of the main migratory flow over Italy during spring, LD migratory birds resulted in providing to the sample a higher contribution (n = 26/43, 60.46%) than SD ones and mainly carried the allochthonous species *H. rufipes*. This result indicates that most of the ticks parasitizing birds arriving in Italy during the study period come from sub-Saharan Africa, and, therefore, they can potentially carry pathogens circulating in those areas. Nevertheless, focusing on the potential contribution in terms of prevalence in infestation rates and in the abundance of ticks, proportionally more SD migrants seem to arrive parasitized to Italy and with a higher tick load than LD ones. This difference may be due to the shorter distance covered and the shorter time taken by SD migrants to reach the Italian peninsula, allowing a higher number of ticks to be still on the host when screened. Despite this prevalence, SD migrants seem to have a marginal contribution in introducing allochthonous ticks to Italy since, in our sample, they were infested only by Western Palearctic ticks already present in the peninsula. Regardless of the migratory strategy, many of the bird species that contribute most to the spread of ticks (prevalence of infestation and tick abundance) seem to share the behavior of feeding (Common redstart, Whinchat, Northern wheatear, Western yellow wagtail, Blackbird, Song thrush, European robin) or resting (Nightjar) on the ground, while for others (e.g., Golden oriole, Chaffinch) it is not immediately clear what conditions may favor tick-bird association. However, this remains a first-glance hypothesis, and other variables must be considered to test the correlation between behavior, habitat, and tick infestation in wintering areas. Additionally, the majority of the infested bird species belong to the Passeriformes order, which is the most represented (40 out of 43 species) and abundant (1061 out of 1079 individuals). A similar result could be explained by the abundance of species within the order and our catching technique specific for small birds. As a consequence, it was expected that the five species (1 SD and 4 LD) providing over 50% of the total number of infested birds are Passeriformes.

In this study, the identification of tick species collected from migratory birds was based on both morphology and molecular tools, and it has been recently detailed by Toma et al. [29]. The incomplete data for the molecular identification of ticks did not allow us to carry out comparative analyses on the rate of infestation by the different parasite species among different host species. However, partial information obtained provided an overall picture of the main tick species introduced to Italy by migratory birds during spring migration.

The presence of African *Amblyomma* ticks, together with the high percentage (>76%) of *Hyalomma* genus ticks (>85% of *H. rufipes*), perfectly match with the sub-Saharan origin of the LD migrants confirming previous findings on Ventotene island [28,30]. Similarly, the presence of the soft tick *Argas vulgaris*, absent in Italy and commonly distributed in southeastern Europe (Russia, Ukraine, Armenia, Israel) and in central and south Asia [42], on an SD migrant, confirms the movement of vectors also within bird’s intra-Western Palearctic routes. 

*H. rufipes* is the most common of its genus in sub-Saharan Africa, and its abundance of bird hosts can explain the increasingly new detections in continental Europe and its naturalization in Asia, North Africa, and other countries of the Mediterranean Basin as a result of dispersion along migratory routes [43,44]. The abundance of *H. rufipes* was expected considering the collection sites. In fact, the detection of allochthonous tick species in the European regions is strictly influenced by the number of species and individuals reaching their breeding grounds at different latitudes and by their migratory strategies. In fact, while central and northern European regions represent the final destination for most of the migratory birds, countries of the Mediterranean Basin represent both transit and temporary stop areas for all the migrants returning to Europe from Africa and, to a lesser extent, breeding quarters for many species. Hence, southernmost European countries are more exposed than central European ones to an extraordinary flow of birds and ticks arriving directly from Africa, as emerged from a five-year study in central Europe [45], where a very low number of LD migrants infested by few ticks of the *H. marginatum* complex were identified. On the contrary, our sampling campaign was characterized by a majority of trans-Saharan migrants carrying mainly *H. rufipes* ticks, almost exclusively distributed in Africa and in Middle Eastern Asia.

High percentages of immature stages of exotic African ticks parasitizing birds during migration and relative pathogenic agents have been reported by several recent studies, mainly in countries of the Mediterranean area [11,26,30,46]. All ticks are obligate parasites of vertebrates, and some species have two hosts during their life cycle, with the larval stage attaching and molting to nymph while still on the same avian host [47]. Nevertheless, some ticks can show a certain flexibility in the number of hosts in particular conditions [48]. Immature stages in species belonging to the *H. marginatum* complex (consisting of *H. isaaci*, *H. marginatum* sensu stricto, *H. rufipes*, *H. turanicum*, and *H. glabrum*) remain on the same avian host up to 26 days before dropping off, molting to adult and search for the second final host [49]. This lapse of time can be sufficient for passerine birds migrating from sub-Saharan Africa to reach Italy and eventually continue their flyways to northern countries [50], causing the dispersion of allochthonous ticks even a thousand kilometers away from the origin areas. The presence of 59.09% of *H. rufipes* nymphs in our study suggests that most of the ticks were probably attached to birds several days before our collection so that they had a time sufficient to develop from larval to nymphal stage. Thus, it is possible that the majority of ticks collected on the Italian islands entered into contact with their hosts in the African wintering quarters immediately before the departure. However, multiple infestations experienced in stop-over sites during migration are possible as suggested by the simultaneous presence of ticks at different life stages (both larvae and nymphs), by the different degree of engorgement of ticks at the same stage, or by the rare contemporary presence of ticks of different species on the bird host. Similarly, episodes of infestation during migratory stop-over in areas of the Western Palearctic are also indicated by the presence of ticks typical of the Mediterranean area on trans-Saharan birds (Figure 2).

The absence of recent evidence of the presence of adults of *H. rufipes* in Italy, where climatic and environmental conditions are expected to be more favorable than in Central and Northern Europe, where this African tick species has been found, is probably due to the lack of studies and active research on livestock and in the environment. The large amount of *Hyalomma* ticks carried by birds also includes a small percentage of *H. marginatum*, carried mainly by SD migrants but also by LD ones, which may have come into contact with these ticks in the stop-over locations along the North African coasts. This species is indeed endemic to almost all of the Mediterranean Basin region, Italy included [51], and some Eastern European countries where it is the most common vector of CCHFV. 

Although different tick species have been demonstrated to be competent vectors for this zoonotic virus, members of the genus *Hyalomma* are considered to be the primary and the most frequent vectors [49,52]. They are two-host parasites, widely distributed in many ecological areas of Palearctic and Afrotropical regions, whose immature stages are often associated with birds [49,53]. Therefore, the marked predominance of ticks of this genus in our sampling indicates that a concrete risk of CCHFV introduction to Italy exists, and it was confirmed by the detection of the viral genome in two ticks collected in two subsequent years, respectively, 2017 and 2018 on the island of Ventotene. The first positivity belongs to an *H. rufipes* nymph collected from a Whinchat [12], the second species among the LD migrants in terms of infestation rate. A larva of *H. rufipes* CCHFV positive in 2018 was collected on a Black-eared wheatear, a trans-Saharan species but one of the less represented in our collection with a few individuals. The presence of the CCHFV in a larval stage confirms the occurrence of transovarial transmission of the virus, as already been experimentally demonstrated in *H. rufipes* [7,8], although with different effectiveness according to the virus strain and tick species [44,54]. The CCHFV detected in 2017 belongs to the genotype Africa III confirming the sub-Saharan origin of the tick [12], but, unfortunately, the scarce viral load in the 2018 tick did not allow any sequencing of the viral strain. These findings in two subsequent years confirm as real the risk of the introduction of CCHFV to Italy by migratory birds arriving from African endemic areas as proposed by Okely et al. [31] in their model. Many countries in the Mediterranean Basin present favorable environmental, climatic, and ecological conditions that allow the diffusion of CCHFV [13], as demonstrated by the recent new establishment of viral circulation in Spain [14]. In Italy, the risk of viral introduction and diffusion is particularly high due to the peculiar position in the middle of the major migratory flyways from Africa and the wild ecological heterogeneity that characterizes its territory. Even though there is no evidence of CCHFV presence in the country, extensive studies on ticks and serological investigation on mammals should be encouraged in those regions where *Hyalomma* vectors have been identified. Unless human hosts are involved in the viraemic cycle showing clinical symptoms, CCHFV circulation can be silent since the infection is asymptomatic in mammals [55]. It was recently confirmed in Corsica, where viral circulation among livestock has been revealed by the presence of specific antibodies without any human cases [56].

WNV and USUV are both maintained in nature by an enzootic cycle involving birds and ornithophilic mosquitoes as species of the *Culex* genus. However, ticks belonging to the *Hyalomma* genus, in which immature stages are strictly associated with birds, have the potential to acquire the virus from an infected host, transmit it to subsequent developmental stages and possibly act as vectors, as demonstrated experimentally [57,58]. Interestingly, WNV can persist in ticks for more than 130 days [57], a very long time if compared to the length of viremia in birds, which enables ticks to potentially disseminate the infection to a large variety of hosts even after several weeks and thousand kilometers attached to a migratory bird. Therefore, our findings of two ticks, identified as *H. marginatum* and *Hyalomma* sp. positive to WNV lineage 1, suggest that WNV can also be spread through ticks carried by birds and livestock and has already emerged in Europe and Africa [59,60]. Lineage 1 has been involved in human infection in Italy since 1998; nevertheless, the evidence of the circulation of WNV lineage 2 goes back to 2011 [61], and now it is the most frequently identified lineage in Italy [62]. Only recently, in 2021, Lineage 1 was found again in 2 birds of prey in Italy but without any evidence of further circulation [63]. Thus, its detection in organs from a dead Garden warbler (LD migrant) suggests the potential role of migratory birds in the spreading of emerging and re-emerging zoonotic pathogens from Africa to Europe.

## 5. Conclusions

Migratory birds can play a crucial role in spreading ticks and pathogens along their flyways, and in particular trans-Saharan migrants can move between continents for several thousand kilometers flying over ecological barriers. Results obtained by our three-year study carried out during spring migration in three islands in Italy confirm how the tick species collected reflect the geographical origin of non-breeding areas of different bird species. Particularly, we observed that the most numerous and parasitized species using these islands as stopovers originate from sub-Saharan Africa and carry immatures of allochthone tick species, mainly belonging to *H. rufipes* species. Conversely, intra-Western Palearctic migratory species are less numerous on these islands, less infested, and mostly parasitized by autochthone ticks of the *Ixodes* genus, including the ones strictly ornithophilic. Moreover, our data demonstrate that dispersion of African ticks can also contribute to the diffusion of new tick-borne zoonotic agents causing diseases representing a threat to human health, such as CCHFV detected in ticks collected from two birds in two subsequent years in Ventotene. However, the absence of infected and exotic species from the ground collection on the stop-over islands [29] indicates that successful colonization of allochthonous ticks or introduction of new tick-borne pathogens is the result of a complex phenomenon depending on many factors such as suitable habitat, climate, and host availability. At the same time, our findings hint that the circulation of WNV in Italy may receive new viral sources every year through migrants, contributing to the introduction of different possible lineages and that ticks can potentially be involved in WNV transmission even though their role in the viral cycle would need further investigation. 

In conclusion, the fundamental role of migratory birds in the introduction and circulation of ticks and tick-borne diseases begins to emerge. Nevertheless, their contribution is still to be defined and needs extensive research focused on understanding how the origin areas, habitat conditions, timing, and behavioral and ecological features of each avian species influence tick-bird interactions and subsequent spread. The risk of introduction and spread of emerging and re-emerging zoonotic diseases represents a threat to human health and highlights the necessity for further investigations and surveillance in Italy and in the Mediterranean Basin, gateways for millions of migratory birds to their breeding grounds every year. 

## Figures and Tables

**Table 1:** Infested bird species and respective tick species.

Common Name	Scientific Name	Migration Strategy	N. Infested Birds	N. Screened Birds	N. Ticks	Prevalence Infested Birds (%)	Abundance of Ticks (%)
European nightjar	*Caprimulgus europaeus*	LD	3	107	25	2.80	23.36
Scops owl	*Otus scops*	LD	2	78	2	2.56	2.56
Eurasian hoopoe	*Upupa epops*	LD	2	91	2	2.20	2.20
Golden oriole	*Oriolus oriolus*	LD	26	418	63	6.22	15.07
Woodchat shrike	*Lanius senator*	LD	6	190	16	3.16	8.42
Icterine warbler	*Hippolais icterina*	LD	60	3937	98	1.52	2.49
Sedge warbler	*Acrocephalus schoenobaenus*	LD	7	343	22	2.04	6.41
Eurasian reed warbler	*Acrocephalus scirpaceus*	LD	7	351	7	1.99	1.99
Great reed warbler	*Acrocephalus arundinaceus*	LD	1	81	3	1.23	3.70
Barn swallow	*Hirundo rustica*	LD	1	818	1	0.12	0.12
Wood warbler	*Phylloscopus sibilatrix*	LD	108	2781	244	3.88	8.77
Willow warbler	*Phylloscopus trochilus*	LD	33	4697	39	0.70	0.83
Radde’s warbler	*Phylloscopus schwarzi*	LD	1	1	1	100.00	100.00
Garden warbler	*Sylvia borin*	LD	16	5181	22	0.31	0.42
Eastern subalpine warbler	*Sylvia cantillans*	LD	12	3818	12	0.31	0.31
Common whitethroat	*Sylvia communis*	LD	88	3779	170	2.33	4.50
Spotted flycatcher	*Muscicapa striata*	LD	86	2180	126	3.94	5.78
Nightingale	*Luscinia megarhynchos*	LD	14	744	28	1.88	3.76
Pied flycatcher	*Ficedula hypoleuca*	LD	83	3563	191	2.33	5.36
Collared flycatcher	*Ficedula albicollis*	LD	8	571	31	1.40	5.43
Common redstart	*Phoenicurus phoenicurus*	LD	121	1745	202	6.93	11.58
Whinchat	*Saxicola rubetra*	LD	79	1210	281	6.53	23.22
Northern wheatear	*Oenanthe oenanthe*	LD	10	227	66	4.41	29.07
Black-eared wheatear	*Oenanthe hispanica*	LD	2	25	6	8.00	24.00
Tree pipit	*Anthus trivialis*	LD	16	618	45	2.59	7.28
Western yellow wagtail	*Motacilla flava*	LD	13	220	25	5.91	11.36
**TOTAL LD**			**805**	**37,774**	**1728**	**2.13**	**4.57**
Great tit	*Parus major*	SD	1	65	1	1.54	1.54
Eurasian skylark	*Alauda arvensis*	SD	1	11	1	9.09	9.09
Chiff chaff	*Phylloscopus collybita*	SD	8	1840	8	0.43	0.43
Blackcap	*Sylvia atricapilla*	SD	3	1292	3	0.23	0.23
Sardinian Warbler	*Sylvia melanocephala*	SD	4	319	4	1.25	1.25
Song thrush	*Turdus philomelos*	SD	42	521	128	8.06	24.57
Blackbird	*Turdus merula*	SD	16	59	31	27.12	52.54
Ring ouzel	*Turdus torquatus*	SD	1	6	5	16.67	83.33
European robin	*Erithacus rubecula*	SD	172	2173	391	7.92	17.99
Black redstart	*Phoenicurus ochruros*	SD	7	322	8	2.17	2.48
Firecrest	*Regulus ignicapilla*	SD	1	48	1	2.08	2.08
Hedge accentor	*Prunella modularis*	SD	8	21	17	38.10	80.95
Chaffinch	*Fringilla coelebs*	SD	4	85	4	4.71	4.71
Hawfinch	*Coccothraustes coccothraustes*	SD	2	30	9	6.67	30.00
Goldfinch	*Carduelis carduelis*	SD	1	88	2	1.14	2.27
Serin	*Serinus serinus*	SD	2	241	2	0.83	0.83
**TOTAL SD**			**273**	**7121**	**615**	**3.83**	**8.64**
Italian sparrow	*Passer italiae*	R	1	152	1	0.66	0.66

Note: LD = long-distance migrant, SD = short-distance migrant, R = resident; prevalence infested birds = N_*Infested*_/N*_screened_* × 100; abundance of ticks = N*_ticks_*/N*s_creened_* × 100.

## Data Availability

Not applicable.

## Supplementary Materials

The following supporting information can be downloaded at: https://www.mdpi.com/article/10.3390/pathogens11091056/s1, Table S1: Raw data of bird–tick association.

## References

[1] Estrada-Peña A., Ostfeld R.S., Peterson A.T., Poulin R., de la Fuente J. (2014). Effects of environmental change on zoonotic disease risk: An ecological primer. Trends Parasitol..

[2] Hasle G. (2013). Transport of ixodid ticks and tick-borne pathogens by migratory birds. Front. Cell. Infect. Microbiol..

[3] Nikolay B. (2015). A review of West Nile and Usutu virus co-circulation in Europe: How much do transmission cycles overlap?. Trans. R Soc. Trop Med. Hyg..

[4] Rizzoli A., Jiménez-Clavero A.M., Barzon L., Cordioli P., Figuerola J., Koraka P., Martina B., Moreno A., Nowotny N., Pardigon N. (2015). The challenge of West Nile virus in Europe: Knowledge gaps and research priorities. Euro Surveill..

[5] World Health Organization (WHO) Annual Review of Diseases Prioritized under the Research and Development Blueprint Informal Consultation 6–7 February 2018, Geneva, Switzerland. https://www.who.int/news-room/events/detail/2018/02/06/default-calendar/2018-annual-review-of-diseases-prioritized-under-the-research-anddevelopment-blueprint.

[6] Mehand M.S., Al-Shorbaji F., Millett P., Murgue B. (2018). The WHO R&D Blueprint: 2018 review of emerging infectious diseases requiring urgent research and development efforts. Antivir. Res..

[7] Zeller H.G., Cornet J.-P., Camicas J.-L. (1994). Experimental Transmission of Crimean-Congo Hemorrhagic Fever Virus by West African Wild Ground-Feeding Birds to Hyalomma marginatum rufipes Ticks. Am. J. Trop. Med. Hyg..

[8] Shepherd A.J., Swanepoel R., Shepherd S.P., Leman P.A., Mathee O. (1991). Viraemic transmission of Crimean-Congo haemorrhagic fever virus to ticks. Epidemiol. Infect..

[9] Palomar A.M., Portillo A., Santibáñez P., Mazuelas D., Arizaga J., Crespo A., Gutiérrez O., Cuadrado J.F., Oteo J.A. (2013). Crimean-Congo Hemorrhagic Fever Virus in Ticks from Migratory Birds, Morocco1. Emerg. Infect. Dis..

[10] Leblebicioglu H., Eroglu C., Erciyas-Yavuz K., Hokelek M., Acici M., Yilmaz H. (2014). Role of migratory birds in spreading Crimean-Congo hemorrhagic fever, Turkey. Emerg Infect. Dis..

[11] Lindeborg M., Barboutis C., Ehrenborg C., Fransson T., Jaenson T.G., Lindgren P.-E., Lundkvist P.E., Nyström F., Salaneck E., Waldenström J. (2012). Migratory Birds, Ticks, and Crimean-Congo Hemorrhagic Fever Virus. Emerg. Infect. Dis..

[12] Mancuso E., Toma L., Polci A., d’Alessio S.G., Di Luca M., Orsini M., Di Domenico M., Marcacci M., Mancini G., Spina F. (2019). Crimean-Congo Hemorrhagic Fever Virus Genome in Tick from Migratory Bird, Italy. Emerg. Infect. Dis..

[13] Estrada-Peña A., Palomar A.M., Santibáñez P., Sánchez N., Habela M.A., Portillo A., Romero L., Oteo J.A. (2012). Crimean-Congo Hemorrhagic Fever Virus in Ticks, Southwestern Europe, 2010. Emerg. Infect. Dis..

[14] Negredo A., Habela M.Á, de Arellano E.R., Diez F., Lasala F., López P., Sarriá A., Labiod N., Calero-Bernal R., Arenas M. (2019). Survey of Crimean‐Congo Hemorrhagic Fever Enzootic Focus, Spain, 2011–2015. Emerg. Infect. Dis..

[15] Ruiz-Fons F., Fernández-De-Mera I.G., Acevedo P., Höfle U., Vicente J., de la Fuente J., Gortazár C. (2006). Ixodid ticks parasitizing Iberian red deer (Cervus elaphus hispanicus) and European wild boar (Sus scrofa) from Spain: Geographical and temporal distribution. Veter.- Parasitol..

[16] Nijhof A.M., Bodaan C., Postigo M., Nieuwenhuijs H., Opsteegh M., Franssen L., Jebbink F., Jongejan F. (2007). Ticks and Associated Pathogens Collected from Domestic Animals in the Netherlands. Vector-Borne Zoonotic Dis..

[17] Hornok S., Horváth G. (2012). First report of adult Hyalomma marginatum rufipes (vector of Crimean-Congo haemorrhagic fever virus) on cattle under a continental climate in Hungary. Parasites Vectors.

[18] Chitimia-Dobler L., Nava S., Bestehorn M., Dobler G., Wölfel S. (2016). First detection of Hyalomma rufipes in Germany. Ticks Tick-Borne Dis..

[19] Chitimia-Dobler L., Schaper S., Rieß R., Bitterwolf K., Frangoulidis D., Bestehorn M., Springer A., Oehme R., Drehmann M., Lindau A. (2019). Imported Hyalomma ticks in Germany in 2018. Parasites Vectors.

[20] Hansford K.M., Carter D., Gillingham E.L., Hernandez-Triana L.M., Chamberlain J., Cull B., McGinley L., Phipps L.P., Medlock J.M. (2019). Hyalomma rufipes on an untraveled horse: Is this the first evidence of Hyalomma nymphs successfully moulting in the United Kingdom?. Ticks Tick-borne Dis..

[21] Grandi G., Chitimia-Dobler L., Choklikitumnuey P., Strube C., Springer A., Albihn A., Jaenson T.G., Omazic A.W. (2020). First records of adult Hyalomma marginatum and H. rufipes ticks (Acari: Ixodidae) in Sweden. Ticks Tick-borne Dis..

[22] Hubálek Z., Sedláček P., Estrada-Peña A., Vojtíšek J., Rudolf I. (2020). First record of Hyalomma rufipes in the Czech Republic, with a review of relevant cases in other parts of Europe. Ticks Tick-borne Dis..

[23] Lesiczka P.M., Daněk O., Modrý D., Hrazdilová K., Votýpka J., Zurek L. (2022). A new report of adult Hyalomma marginatum and Hyalomma rufipes in the Czech Republic. Ticks Tick-borne Dis..

[24] Cicculli V., de Lamballerie X., Charrel R., Falchi A. (2019). First molecular detection of Rickettsia africae in a tropical bont tick, Amblyomma variegatum, collected in Corsica, France. Exp. Appl. Acarol..

[25] Pintore E., Olivieri E., Floriano A.M., Sassera D., Sanna N., Garippa G. (2020). First detection of Amblyomma variegatum and molecular finding of Rickettsia africae in Sardinia, Italy. Ticks Tick-Borne Dis..

[26] Hoffman T., Carra L.G., Öhagen P., Fransson T., Barboutis C., Piacentini D., Figuerola J., Kiat Y., Onrubia A., Jaenson T.G. (2021). Association between guilds of birds in the African-Western Palaearctic region and the tick species Hyalomma rufipes, one of the main vectors of Crimean-Congo hemorrhagic fever virus. One Health.

[27] Rollins R.E., Schaper S., Kahlhofer C., Frangoulidis D., Strauß A.F., Cardinale M., Springer A., Strube C., Bakkes D.K., Becker N.S. (2021). Ticks (Acari: Ixodidae) on birds migrating to the island of Ponza, Italy, and the tick-borne pathogens they carry. Ticks Tick-Borne Dis..

[28] Toma L., Mancini F., Di Luca M., Cecere J.G., Bianchi R., Khoury C., Quarchioni E., Manzia F., Rezza G., Ciervo A. (2014). Detection of Microbial Agents in Ticks Collected from Migratory Birds in Central Italy. Vector-Borne Zoonotic Dis..

[29] Toma L., Mancuso E., d’Alessio S.G., Menegon M., Spina F., Pascucci I., Monaco F., Goffredo M., Di Luca M. (2021). Tick species from Africa by migratory birds: A 3-year study in Italy. Exp. Appl. Acarol..

[30] Pascucci I., Di Domenico M., Capobianco Dondona G., Di Gennaro A., Polci A., Capobianco Dondona A., Mancuso E., Cammà C., Savini G., Cecere J.G. (2019). Assessing the role of migratory birds in the introduction of ticks and tick-borne pathogens from African countries: An Italian experience. Ticks Tick-Borne Dis..

[31] Okely M., Anan R., Gad-Allah S., Samy A. (2020). Mapping the environmental suitability of etiological agent and tick vectors of Crimean-Congo hemorrhagic fever. Acta Trop..

[32] Hoffman T., Lindeborg M., Barboutis C., Erciyas-Yavuz K., Evander M., Fransson T., Figuerola J., Jaenson T.G., Kiat Y., Lindgren P.-E. (2018). Alkhurma Hemorrhagic Fever Virus RNA in *Hyalomma rufipes* Ticks Infesting Migratory Birds, Europe and Asia Minor. Emerg. Infect. Dis..

[33] Spina F., Massi A., Montemaggiori A., Baccetti N. (1993). Spring migration across central Mediterranean: General results from the “Progetto Piccole Isole”. Die Vogelwarte.

[34] Tenan S., Spina F. (2010). Timing and condition-related effects on recapture probability, mass change and stopover length of spring migrating songbirds on A small Mediterranean island. Ardeola.

[35] Newton I. (2010). The Migration Ecology of Birds.

[36] Manilla G. (1998). Fauna d’Italia—Ixodida.

[37] Iori A., Di Giulio A., De Felici S. (2005). Zecche d’Italia parte III. Mappe Parassitologiche.

[38] Wölfel R., Paweska J.T., Petersen N., Grobbelaar A.A., Leman P.A., Hewson R., Georges-Courbot M.-C., Papa A., Günther S., Drosten C. (2007). Virus Detection and Monitoring of Viral Load in Crimean-Congo Hemorrhagic Fever Virus Patients. Emerg. Infect. Dis..

[39] Del Amo J., Sotelo E., Fernández-Pinero J., Gallardo C., Llorente F., Agüero M., Jiménez-Clavero M.A. (2013). A novel quantitative multiplex real-time RT-PCR for the simultaneous detection and differentiation of West Nile virus lineages 1 and 2, and of Usutu virus. J. Virol. Methods.

[40] Vázquez A., Herrero L., Negredo A., Hernández L., Sánchez-Seco M.P., Tenorio A. (2016). Real time PCR assay for detection of all known lineages of West Nile virus. J. Virol. Methods.

[41] Cavrini F., DELLA Pepa M.E., Gaibani P., Pierro A.M., Rossini G., Landini M.P., Sambri V. (2011). A rapid and specific real-time RT-PCR assay to identify Usutu virus in human plasma, serum, and cerebrospinal fluid. J. Clin. Virol..

[42] Dusbábek F. (1976). Argas (Argas) vulgaris Filippova, 1961, a new member of Czechoslovak tick fauna. Folia Parasitol..

[43] Bakirci S., Sarali H., Aydin L., Eren H., Karagenc T., Bakırcı S. (2012). Distribution and seasonal activity of tick species on cattle in the West Aegean region of Turkey. Exp. Appl. Acarol..

[44] EFSA Panel On Animal Health and Welfare (AHAW) (2010). Scientific Opinion on Geographic Distribution of Tick-borne Infections and their Vectors in Europe and the other Regions of the Mediterranean Basin. EFSA J..

[45] Capek M., Literak I., Kocianova E., Sychra O., Najer T., Trnka A., Kverek P. (2014). Ticks of the Hyalomma marginatum complex transported by migratory birds into Central Europe. Ticks Tick-Borne Dis..

[46] De Liberato C., Frontoso R., Magliano A., Montemaggiori A., Autorino G.L., Sala M., Bosworth A., Scicluna M.T. (2018). Monitoring for the possible introduction of Crimean Congo haemorrhagic fever virus in Italy based on tick sampling on migratory birds and serological survey of sheep flocks. Prevent. Vet. Med..

[47] Walker A.R., Bouattour A., Camicas J.-L., Estrada-Peña A., Horak I.G., Latif A.A., Pegram R.G., Preston P.M. (2003). Ticks of Domestic Animals in Africa: A Guide to Identification of Species.

[48] Farkas R., Estrada-Peña A., Jaenson T.G.T., Pascucci I., Madder M., Salman M., Tarrés-Call J. (2012). Ticks and Tick-Borne Diseases: Geographical Distribution and Control Strategies in the Euro-Asia Region.

[49] Hoogstraal H. (1979). Review Article 1: The Epidemiology of Tick-Borne Crimean-Congo Hemorrhagic Fever in Asia, Europe, and Africa. J. Med. Entomol..

[50] Newton I. (2007). The Migration Ecology of Birds.

[51] European Centre for Disease Prevention and Control and European Food Safety Authority (2022). Tick Maps [Internet]. Stockholm: ECDC. https://ecdc.europa.eu/en/disease-vectors/surveillance-and-disease-data/tick-maps.

[52] Gargili A., Estrada-Peña A., Spengler J.R., Lukashev A., Nuttall P.A., Bente D.A. (2017). The role of ticks in the maintenance and transmission of Crimean-Congo hemorrhagic fever virus: A review of published field and laboratory studies. Antivir. Res..

[53] Spengler J.R., Estrada-Peña A. (2018). Host preferences support the prominent role of Hyalomma ticks in the ecology of Crimean-Congo hemorrhagic fever. PLOS Neglected Trop. Dis..

[54] Estrada-Peña A., Farkas R., Jaenson T.G., Madder M., Pascucci I., Tarrés-Call J. (2010). Scientific opinion on the role of tick vectors in the epidemiology of Crimean-Congo hemorrhagic fever and African swine fever in Eurasia: EFSA Panel on Animal Health and Welfare. EFSA J..

[55] Spengler J.R., Estrada-Peña A., Garrison A.R., Schmaljohn C., Spiropoulou C.F., Bergeron É., Bente D.A. (2016). A chronological review of experimental infection studies of the role of wild animals and livestock in the maintenance and transmission of Crimean-Congo hemorrhagic fever virus. Antivir. Res..

[56] Grech-Angelini S., Lancelot R., Ferraris O., Peyrefitte C.N., Vachiery N., Pédarrieu A., Peyraud A., Rodrigues V., Bastron D., Libeau G. (2020). Crimean-Congo Hemorrhagic Fever Virus Antibodies among Livestock on Corsica, France, 2014–2016. Emerg. Infect. Dis..

[57] Formosinho P., Santos-Silva M.M. (2006). Experimental infection of Hyalomma marginatum ticks with West Nile virus. Acta Virol..

[58] Lawrie C.H., Uzcátegui N.Y., Gould E.A., Nuttall P. (2004). Ixodid and Argasid Tick Species and West Nile Virus. Emerg. Infect. Dis..

[59] Kolodziejek J., Marinov M., Kiss B.J., Alexe V., Nowotny N. (2014). The Complete Sequence of a West Nile Virus Lineage 2 Strain Detected in a Hyalomma marginatum marginatum Tick Collected from a Song Thrush (Turdus philomelos) in Eastern Romania in 2013 Revealed Closest Genetic Relationship to Strain Volgograd 2007. PLoS ONE.

[60] Lwande O.W., Venter M., Lutomiah J., Michuki G., Rumberia C., Gakuya F., Obanda V., Tigoi C., Odhiambo C., Nindo F. (2014). Whole genome phylogenetic investigation of a West Nile virus strain isolated from a tick sampled from livestock in north eastern Kenya. Parasites Vectors.

[61] Savini G., Capelli G., Monaco F., Polci A., Russo F., Di Gennaro A., Marini V., Teodori L., Montarsi F., Pinoni C. (2012). Evidence of West Nile virus lineage 2 circulation in Northern Italy. Veter. Microbiol..

[62] Zehender G., Veo C., Ebranati E., Carta V., Rovida F., Percivalle E., Moreno A., Lelli D., Calzolari M., Lavazza A. (2017). Reconstructing the recent West Nile virus lineage 2 epidemic in Europe and Italy using discrete and continuous phylogeography. PLoS ONE.

[63] Mencattelli G., Iapaolo F., Monaco F., Fusco G., de Martinis C., Portanti O., Di Gennaro A., Curini V., Polci A., Berjaoui S. (2021). West Nile Virus Lineage 1 in Italy: Newly Introduced or a Re-Occurrence of a Previously Circulating Strain?. Viruses.

