# Supplementary material for: Direct and Indirect Role of Migratory Birds in Spreading CCHFV and WNV: A Multidisciplinary Study on Three Stop-Over Islands in Italy
Source: Pathogens. 2022 Sep 16;11(9):1056. doi: 10.3390/pathogens11091056 (PMC9505975; doi:10.3390/pathogens11091056)
Supplement: Supplementary file 1 [file pathogens-11-01056-s001.zip › pathogens-1857638-supplementary/Table S1.pdf]

Table S1. Raw data of bird-tick association

	Common name	Sub-Saharan ticks (n)				Western-Palearctic ticks (n)								Undefined origin (n)		
		Amblyomma marmoreum	*Amblyomma variegatum*	*Hyalomma rufipes*	*Hyalomma truncatum*	*Hyalomma marginatum*	*Ixodes ricinus*	*Ixodes frontalis*	*Ixodes ventalloi*	*Ixodes inopinatus*	*Haemaphysalis punctata*	*Haemaphysalis erinacei*	*Argas vulgaris*	*Hyalomma* sp.	*Ixodes* sp.	*Haemaphysalis* sp.
LD	European nightjar			1										24		
	Scops owl					1								1		
	Eurasian hoopoe													2		
	Golden oriol			28		1								34		
	Woodchat shrike			4										12		
	Icterine warbler	3		25		1	1						1	67		
	Sedge warbler			2										20		
	Eurasian reed warbler			5										2		
	Great reed warbler			3												
	Barn swallow			1												
	Wood warbler			83		2								159		
	Willow warbler	2		11					1					24	1	
	Radde's warbler							1								
	Garden warbler			10				1						11		
	Eastern subalpine warbler			5			1	2	2	1				0	1	
	Common whitethroat			52		2	2	2	1					109	2	
	Spotted flycatcher	2		39		1		1						83	1	
	Nightingale			10		2	1							15		
	Pied flycatcher			52				1						138		
	Collared flycatcher			4										27		
	Common redstart			65	2	4	2	2						122		
	Whinchat	1		65										214	1	
	Northern wheathear			7										59		
	Black-eared wheatear			2										4		

	Tree pipit	5	1	3				1						34		1
	Western yellow wagtail			11										13		1
SD	Great tit													1		
	Eurasian skylark								1							
	Chiff chaff						2	5						1		
	Blackcap							2							1	
	Sardinian warbler							3	1							
	Song thrush			2		57	2	17	4					28	19	
	Blackbird						4	13	3						11	
	Ring ouzel								5							
	European robin					22	17	102	22					3	227	
	Black redstart			1				2	2		1	2	1			
	Firecrest														1	
	Hedge accentor					4	1		6						6	
	Chaffinch							2	1						1	
	Hawfinch						1			1					7	
	Goldfinch					1								1		
	Serin							1	1							
R	Italian sparrow							1								
	**TOTAL**	13	1	491	2	98	34	159	50	2	1	2	2	1208	279	2

Note: LD=long-distance migrant, SD=short-distance migrant, R=resident
